# Molecular Orientation of a Terbium(III)-Phthalocyaninato Double-Decker Complex for Effective Suppression of Quantum Tunneling of the Magnetization

**DOI:** 10.3390/molecules22060999

**Published:** 2017-06-15

**Authors:** Tsutomu Yamabayashi, Keiichi Katoh, Brian K. Breedlove, Masahiro Yamashita

**Affiliations:** 1 Department of Chemistry, Graduate School of Science, Tohoku University, 6-3, Aramaki-Aza-Aoba, Aoba-ku, Sendai, Miyagi 980-8578, Japan; tsutomu.yamabayashi.p3@dc.tohoku.ac.jp (T.Y.); breedlove@m.tohoku.ac.jp (B.K.B.); 2 WPI Research Center, Advanced Institute for Materials Research, Tohoku University, 2-1-1 Katahira, Aoba-ku, Sendai 980-8577, Japan; 3 School of Materials Science and Engineering, Nankai University, Tianjin 300350, China

**Keywords:** single-molecule magnets, terbium(III)-phthalocyaninato double-decker complex, quantum tunneling of magnetization, magnetic dipole-dipole interaction

## Abstract

Single-molecule magnet (SMM) properties of crystals of a terbium(III)-phthalocyaninato double-decker complex with different molecular packings (**1**: TbPc_2_, **2**: TbPc_2_·CH_2_Cl_2_) were studied to elucidate the relationship between the molecular packing and SMM properties. From single crystal X-ray analyses, the high symmetry of the coordination environment of **2** suggested that the SMM properties were improved. Furthermore, the shorter intermolecular Tb–Tb distance and relative collinear alignment of the magnetic dipole in **2** indicated that the magnetic dipole–dipole interactions were stronger than those in **1**. This was confirmed by using direct current magnetic measurements. From alternating current magnetic measurements, the activation energy for spin reversal for **1** and **2** were similar. However, the relaxation time for **2** is three orders of magnitude slower than that for **1** in the low-*T* region due to effective suppression of the quantum tunneling of the magnetization. These results suggest that the SMM properties of TbPc_2_ highly depend on the molecular packing.

## 1. Introduction

Single-molecule magnets (SMMs) have interesting quantum properties, such as slow magnetic relaxation [1,2] and quantum tunneling of magnetization (QTM) [3,4,5]. Since the discovery of the Mn_12_ cluster, several compounds showing slow magnetic relaxation have been reported. Lanthanoid(III) (Ln^III^) complexes have been extensively studied because Ln^III^ ions have a ground state multiplet with large angular momenta, *J* = *L* + *S*, and ligand field (LF) splitting of the ground state gives a large activation energy for spin reversal (*U*_eff_) compared to polynuclear complexes of transition metal ions [6,7,8,9].

One of the most promising classes of Ln SMMs is a family of bisphthalocyaninato complexes (LnPc_2_) [8,9,10,11,12,13,14,15,16], which were shown to be SMMs in 2003 [8]. The electronic structure of LnPc_2_ has been reported by Ishikawa and co-workers [10]. The ground state of the Tb^III^ ions, ^7^*F*_6_, which is caused by Russell–Saunders coupling, is mainly split by the strong axial LF around the Tb^III^ ion. As a result, there is an energy gap between the ground states with *J*_z_ = ±6 and the first excited states with *J*_z_ = ±5 of ~400 cm^−1^, which is attributed to *U*_eff_. Due to such a large *U*_eff_, TbPc_2_ shows slow magnetic relaxation up to ~50 K, far surpassing transition metal SMMs, like the Mn_12_ cluster (~4 K). In addition to the high *U*_eff_ value, the high chemical stability and flat shape of the Pc ligands of TbPc_2_ molecule enable it to be exploited in spintronics devices, such as spin transistors [17], spin valves [18,19] and spin quantum bits [20]. In those applications, quantum phenomena, such as QTM, are used to manipulate the spin states. For example, in the research on spin transistors, TbPc_2_ molecules have been inserted between gold electrodes, and addressing and detecting single nuclear spin states of the Tb ion have been demonstrated by using QTM events, which cause an abrupt jump in the differential conductance, d*I*/d*V* [17].

On the other hand, nobody has prepared a quantum memory device based on TbPc_2_ since TbPc_2_ shows magnetic hysteresis only below 2 K despite the large *U*_eff_ value [10,11,21]. This is mainly due to QTM, which takes place at random between the energetically matched levels on the opposite sides of the barrier. In 2013, Sessoli et al. reported that the magnetic hysteresis of TbPc_2_ depended on the environment of the crystalline phase [21]. They report that a thermally treated amorphous sample does not show magnetic hysteresis even at 2 K, whereas a pristine crystalline sample shows clear hysteresis at the same temperature. The disappearance of the hysteresis is not due to the degradation of the material but to a significant increase in the QTM rate, which they confirm by using alternating current (ac) magnetic susceptibility measurements. They conclude that transverse terms of the magnetic anisotropy, which accelerate the QTM rate, are induced by the different crystal packing environments in the amorphous samples.

The relationship between coordination geometry and LF parameters in the Hamiltonian have been extensively studied both experimentally and theoretically [8,9,10,11,12,13,14,15,16,22,23,24,25,26,27,28]. These studies show that *D*_4d_ symmetry of the coordination geometry of Ln^III^ ions leads to quenching the off-diagonal term, which contributes to transverse anisotropy. Recently, our group has reported that the closer the twist angle between ligands (*ϕ*) is to 45°, the greater the *U*_eff_ value, and this can be adjusted by fine tuning the octa-coordination geometries using a combination of porphyrin and phthalocyanine ligands [29]. This result is consistent with the fact that the contribution of the off-diagonal LF terms is due to the symmetry of the octa-coordination environment.

In addition to the LF parameters, in a recent study, it has been shown that Ln–Ln interactions, so-called f–f interactions, have a large effect on the SMM properties in the solid state [22,30,31,32,33,34,35,36,37,38,39,40,41,42,43]. In Ln SMMs, 4f electrons, which are responsible for the magnetism, are strongly shielded by the outer shell electrons. Therefore, the exchange interactions via overlap of the 4f orbits are negligibly small, and the magnetic dipole-dipole (MD) interactions are the dominant intermolecular interactions [32,34]. The MD interactions are known to act as an internal magnetic field [38]. In applied direct current (dc) fields, the energies of the up and down spin states of SMMs become different due to Zeeman splitting. As a result, applied dc fields diminish QTM between ground states, and the relaxation time (*τ*) increases. On the other hand, when transverse fields are applied, the ground and excited states mix, inducing QTM [44,45]. Since the magnetic field made by the magnetic moment of SMMs is highly anisotropic, the direction of the easy magnetization between the Ln ions heavily affects the SMM properties. In other words, when the easy axes of the magnetization of two SMMs align collinearly, *τ* increases due to the suppression of QTM, and SMM properties improve. In contrast, if the easy axes do not orientate in the same direction, QTM is enhanced, and SMM properties degrade [46].

As mentioned above, the SMM properties are strongly affected by QTM when the environment is different from the crystalline phase. Considering two components of the LF parameters and the MD interactions, we focused on two crystal structures of TbPc_2_ [47,48] with or without crystal solvent molecules. In this study, we compared the molecular structure and the spin relaxation dynamics, and herein we present an effective molecular design strategy for suppressing QTM via the coordination geometry and the MD interactions.

## 2. Results and Discussion

### 2.1. Comparison of the Crystal Structures of ***1*** and ***2***

**Figure 1:**
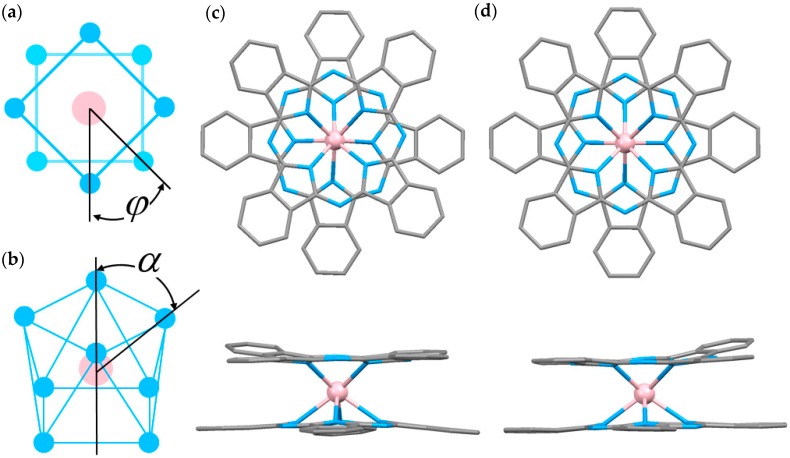
(**a**) Twist angle in square antiprism (SAP) in LnPc_2_; (**b**) Schematic illustration of the SAP coordination environment of LnPc_2_. Crystal structures of **1** (**c**) and **2** (**d**). Top view (upper) and side view (lower). Hydrogen atoms were omitted for clarity. (Tb, pink; N, blue; C, gray).

**Figure 2:**
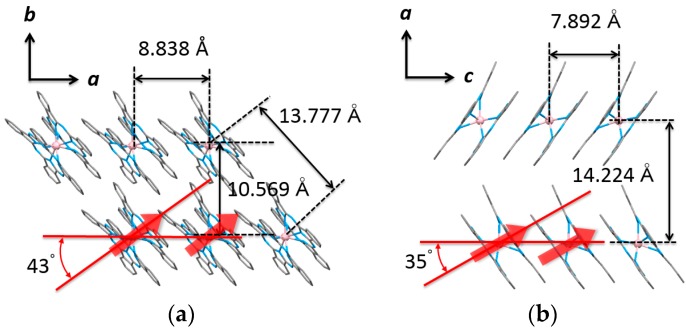
Molecular packing of TbPc_2_ (**a**) for **1** viewed from the *c* axis, (**b**) for **2** viewed from the *b* axis. The values in the figure are the intermolecular Tb^III^–Tb^III^ distances. Hydrogen atoms were omitted for clarity.

TbPc_2_ crystallized without any crystal solvent molecules giving **1** and with dichloromethane molecules giving **2** as reported previously [47,48] in the orthorhombic space groups *P*2_1_2_1_2_1_ and *Pnma*, respectively (Figure 1). The average distance between the Tb^III^ ions and a coordinated isoindole N atom (N_iso_) was determined to be 2.408 Å in **1** and 2.418 Å in **2**. The twist angle (*ϕ*) between the two Pc rings was determined to be 41.37° in **1** and 44.93° in **2**, causing a square antiprism (SAP) coordination geometry and a pseudo four-fold axis (*C*_4_) perpendicular to the Pc rings in both crystal structures. TbPc_2_ has a magnetic easy axis in same orientation with the *C*_4_ axis, as shown in Figure 2 with the red arrow. In addition, the angle (*α*) between the *C*_4_ axis and the direction of the Ln^III^–N_iso_ coordination bond is known to have a strong influence on the LF parameters [22]. It was 54.56° in **1** and 54.60° in **2**.

The LF Hamiltonian can be written as ${\hat{H}}_{\mathrm{LF}}=\sum_{k\;=2,4,6}\sum_{q=-k}^{k}B_{k}^{q}O_{k}^{q}$. $B_{k}^{q}$ is LF parameters, where *q* accounts for the proportionality between the electrostatic potential, *k* is the order of spherical harmonicity, and $O_{k}^{q}$ are spin operators [22,23]. For ideal *D*_4d_ SAP symmetry (*ϕ* = 45° and *α* = 54.74°), only three parameters (*k* = 2, 4, and 6; *q* = 0) are needed, and these parameters contribute to the axial anisotropy. When coordination geometry is distorted from ideal *D*_4d_, the off-diagonal terms (*B*_4_^4^,*B*_6_^4^) , which are parameters for the transverse anisotropy, appear in the Hamiltonian. They cause mixing between the ground states of the up and down spins and induce QTM. As describe above, *ϕ* strongly affects the SMM properties via the LF parameters since the structures deviate from *D*_4d_ symmetry. In this study, the deviation from *D*_4d_ symmetry is smaller for **2** than it is for **1**. Therefore, we think that QTM in **2** is effectively suppressed.

π–π stacking between the intermolecular Pc ligands caused a slipped column structure in both. The strength of the MD interactions is inversely proportional to one third the distance between spin *i* and *j* (*r*_ij_). The nearest Tb^III^–Tb^III^ distance was determined to be 8.838 Å in **1** and 7.892 Å in **2**. Moreover, the strength of the dipole interactions depend on the quantity (3*cos*^2^*θ* – 1), where *θ* is angle made by the magnetic easy axis and the line between neighboring Tb^III^ ions in the same column. *θ* is 43° in **1** and 35° in **2**. Because the *θ* values are less than 54.7°, the so-called magic angle, we thought that ferromagnetic MD interactions were active in both **1** and **2** [49] and that the MD interactions were stronger in **2** than they were in **1**. Selected crystallographic data for **1** and **2** are compiled in Table 1.

### 2.2. Static Magnetic Properties

**Figure 3:**
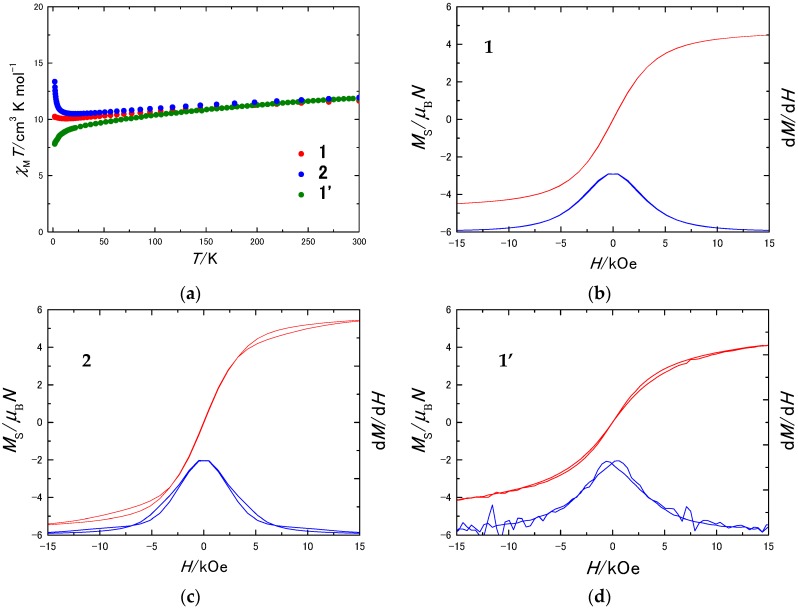
(**a**) Direct current (dc) magnetic susceptibility for **1**, **2**, and **1′**. The solid lines are guides for eyes. Magnetization (*M*) versus field (*H*) and d*M*/d*H* versus *H* for (**b**) **1**; (**c**) **2**; and (**d**) **1′** at 1.82 K. Average field sweep rate was 25 Oe s^−1^.

To determine the magnetic interactions in each molecular packing, dc magnetic measurements were performed. To eliminate the effects of intermolecular interactions, magnetically diluted crystalline samples were prepared (**1′**) by doping TbPc_2_ into YPc_2_, of which the crystal is isomorphous with that of **1**. Both TbPc_2_ and YPc_2_ have an unpaired electron delocalized on the Pc ligands [50,51]. Exchange interactions mediated by π-stacking of Pc ligands in YPc_2_ compounds, where MD interactions are negligible compared to TbPc_2_ have been extensively investigated. Literature reports on the low *T* behavior of YPc_2_ indicate that antiferromagnetic interactions are active along the chains of stacked YPc_2_. In contrast, YPc_2_·CH_2_Cl_2_ exhibits ferromagnetic interactions along the stacked chain [51,52]. As shown in Figure 3a, the *χ*_M_*T* values for **1** and **2** increased with a decrease in *T* below 10 K due to ferromagnetic MD interactions between the Tb^III^ ions. The increase is larger for **2** than it is for **1**. This result indicates that the MD interactions in **2** are stronger, which is consistent with the conclusions from the crystal structure. In contrast, the *χ*_M_*T* value for **1′** decreased with a decrease in *T* because of depopulation of the excited states [53,54]. In addition, our observations suggest that exchange interactions in TbPc_2_ compounds are negligibly small compared to the MD interactions.

In the magnetization (*M*) versus field (*H*) curves for **1**, **2**, and **1′** at 1.82 K, magnetic hysteresis was observed. The area inside the loop increased in order of **1**, **1′**, and **2**. This result shows that the MD interactions affect the magnetic hysteresis. On the other hand, the magnetic isolation of TbPc_2_ to minimize the MD interactions also improved the SMM properties, as previously reported for most SMMs [36,55,56,57,58,59]. Therefore, we concluded that the MD interactions in **1** degraded the SMM properties.

### 2.3. Dynamic Magnetic Properties

**Figure 4:**
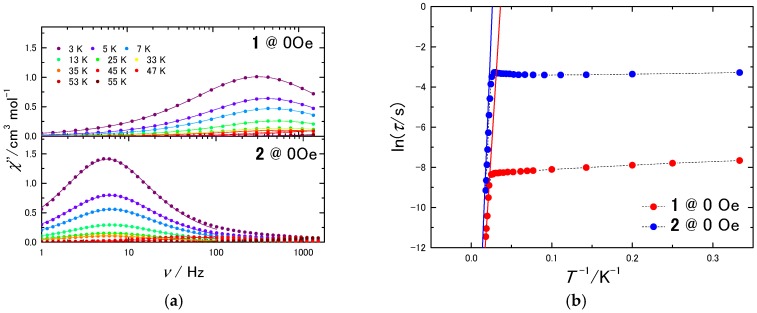
(**a**) *χ*_M_” vs. *ν* plot for **1** and **2** in a zero field. The solid lines were fitted by using the generalized Debye model; (**b**) Arrhenius plots for **1** and **2**. The solid lines were fitted by using the Arrhenius equation. The dashed lines are guides for eyes.

To investigate the magnetic relaxation process, ac magnetic measurements were performed on **1** and **2** with and without an applied external magnetic field (*H*_dc_). *τ* was obtained by simultaneously fitting the real (*χ*_M_’) and imaginary (*χ*_M_”) parts of the ac magnetic susceptibility with the generalized Debye model (Equations (S1) and (S2)) [60]. The peaks in *χ*_M_” plot for **2** were observed in a lower frequency (*ν*) region than they were for **1**, meaning that *τ* was slower for **2**. As seen in Figure 4b, the plot is divided into two parts. In the high-*T* region, where *τ* depends on *T*, the Orbach process is dominant [61]. *U*_eff_ and frequency factor (*τ*_0_) were determined by fitting the data in the high-*T* region using the Arrhenius equation (Equation (S6)) (**1**; *U*_eff_ = 523 cm^−1^, *τ*_0_ = 7.7 × 10^−12^ s, **2**; *U*_eff_ = 556 cm^−1^, *τ*_0_ = 2.2 × 10^−10^ s). Although in the low-*T* region, we tried to fit *τ* for **1**, which still has some dependence on *T*, by using combinations of direct, Raman, and QTM relaxation processes, the data could not be correctly fit (Figure S7 direct + QTM, Figure S8 Raman + QTM, Figure S9 direct + Raman + QTM). We think that intermolecular interactions affect the spin ground state as a perturbation and induce complex mixing of the relaxation process. We could fit the data points for **1** in the low-*T* region by considering the Orbach process and QTM (*U*_eff_ = 3.92 cm^−1^, *τ*_0_ = 3.3 × 10^−4^ s, *τ*_QTM_ = 7.84 × 10^−4^ s) (Figure S10), supporting that relaxation occurs through a complex mixture of processes in the low-*T* region. One TbPc_2_ molecule does not have such an excited spin state *U*_eff_ = 3.92 cm^−1^ for the Orbach process, whereas in the crystal structure, intermolecular magnetic interactions can split the ground state as reported for the Tb triple-decker complex [37]. On the other hand, *τ* for **2**, which scarcely depends on *T*, was fitted by considering QTM (*τ*_QTM_ = 3.51 × 10^−2^ s) (Figure S11). *U*_eff_ values for **1** and **2** in the high-*T* region were found to be similar. However, the *τ* values were different in the low-*T* region.

**Figure 5:**
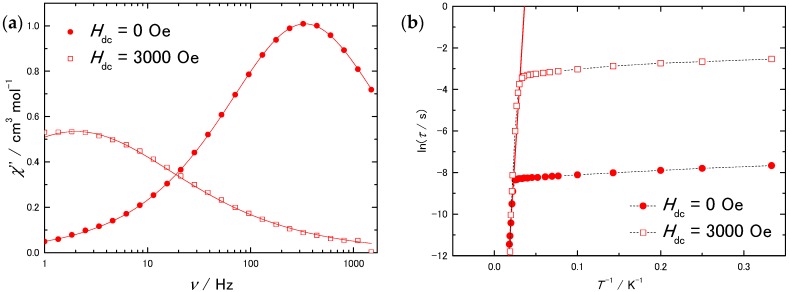
(**a**) *χ*_M_” vs. *ν* plot for **1** in *H*_dc_ of 0 Oe and 3000 Oe at 3 K. The solid lines were fitted by using the generalized Debye model. (**b**) Arrhenius plots for **1** in *H*_dc_ of 0 Oe and 3000 Oe. The solid lines were fitted by using the Arrhenius equation. The dashed lines are guides for eyes.

In an *H*_dc_ of 3000 Oe, the peak in *χ*_M_” plot for **1** clearly shifted toward the low *ν* region, as shown in Figure 5a. Arrhenius plot for **1** in *H*_dc_ of 3000 Oe (Figure 5b) was fitted by using the Arrhenius equation for high-*T* region (*U*_eff_ = 512 cm^−1^, *τ*_0_ = 5.3 × 10^−12^ s) and a combination of the Orbach process and QTM for the low-*T* region (*U*_eff_ = 9.61 cm^−1^, *τ*_0_ = 4.2 × 10^−2^ s, *τ*_QTM_ = 8.83 × 10^−2^ s) (Figure S12). The *H*_dc_ did not affect *U*_eff_ for the high-*T* region. On the other hand, *H*_dc_ caused *τ* to be three orders of magnitude longer than it was in an *H*_dc_ of 0 Oe. Since the *H*_dc_ induce Zeeman splitting, which causes a difference in the energies of the spin states, the QTM rate between ground states was slower, and *τ* increased.

These results show that the molecular packing in **2** effectively suppresses QTM via the small contributions of the off-diagonal terms in the LF Hamiltonian and the relatively strong MD interactions. On the other hand, although ferromagnetic MD interactions were active in **1**, the *τ* values in low-*T* region were similar to those for **1′** (*τ* ≈ 10^−4^ s). This indicates that the MD interactions in **1** do not suppress QTM. We believe that this is because of the large *θ* value mentioned in crystal structure section. Moreover, from the results of dc and ac magnetic measurements, not only the off-diagonal terms but also the collinearity of the MD interactions strongly affect QTM.

## 3. Materials and Methods

### 3.1. Preparation of TbPc_2_ (***1***) and TbPc_2_·CH_2_Cl_2_ (***2***)

TbPc_2_ was synthesized following a reported procedure [47,48]. The obtained powder sample was recrystallized from CHCl_3_/MeOH, which afforded deep green needle-like crystals of **1**, and recrystallized from CH_2_Cl_2_/Hexane, which afforded deep green needle-like crystals of **2**.

### 3.2. Preparation of Magnetically Diluted Sample (***1′***)

TbPc_2_ 5.69 mg (4.04 mmol) and YPc_2_ 49.54 mg (37.01 mmol) were mixed in 10 mL of CHCl_3_ by using ultrasonication (Bransonic^®^ ultrasonic cleaner 2510MT, Bransonic Ultrasonics Corporation, Danbury, CT, USA) for 1 h. Addition of an excess amount of hexane afforded a powder sample of diluted TbPc_2_ (**1′**).

### 3.3. Physical Property Measurements

Powder X-ray diffraction (PXRD) measurements were performed on crushed polycrystalline samples by using an AFC-7R/ LW (Rigaku, Akishima, Japan) operated at 50 kV and 300 mA at 293 K (Figure S1). The data were collected in the diffraction angle range of 3–60° in steps of 0.02° every 2 s. To prevent the crystal solvent from evaporating, the samples were loaded into a capillary (diameter: 0.8 mm, length: 80 mm, Hilgenderg GmbH, Malsfeld, Germany) with the mother liquor. PXRD patterns were simulated from the single-crystal data by using Mercury 3.0 (The Cambridge Crystallographic Data Centre, Cambridge, UK).

Magnetic susceptibility measurements were performed by using Quantum Design SQUID magnetometer (MPMS-XL and MPMS-3, Quantum Design, Inc., San Diego, CA, USA). Direct current measurements were performed in the *T* range of 1.8–300 K in dc magnetic fields (*H*_dc_) of −70.000 to 70,000 Oe. Alternating current measurements were performed in the frequency (*ν*) range of 1–1488 Hz in an *H*_ac_ of 3 Oe in the presence of an *H*_dc_ (zero and 3000 Oe). Measurements were performed on randomly oriented powder samples of **1** and **2**, which were placed in gel capsules and fixed with *n*-eicosane to prevent them from moving during measurements. All data were corrected for *n*-eicosane and diamagnetic contribution from the molecules by using Pascal’s constants. 

## 4. Conclusions

In this work, we synthesized two different crystals of a terbium(III)-phthalocyaninato double-decker complex (**1**: TbPc_2_, **2**: TbPc_2_·CH_2_Cl_2_) and investigated the relationship between molecular packing and magnetic properties. From crystal structure analysis, the *ϕ* value near 45° for **2** corresponded to a small contribution of the off-diagonal terms in the LF Hamiltonian. In addition, the nearest Tb^III^–Tb^III^ distance is shorter, and the TbPc_2_ molecules packed with a small *θ* for **2**, suggesting that MD interactions are stronger in **2** than they are in **1**. This is consistent with the results obtained from dc magnetic measurements. *τ* of **2** exhibited similar behavior with that of **1** in an *H*_dc_ of 3000 Oe, and they were relatively slow. These results suggest that the molecular packing in **2** is suitable for suppressing QTM. In contrast, although ferromagnetic MD interactions are active in **1**, the *τ* values were similar to those of **1′** where no magnetic interactions occur. From these results, we concluded that the collinearity of the MD interactions was important for suppressing QTM. We believe that we can increase *τ* by properly tuning the three parameters *ϕ*, *r*_ij_, *θ*, and this idea can be applied to the design of SMMs with slow *τ*.

## Figures and Tables

**Table 1:** Structural parameters for **1** and **2**.

	1	2
average Tb–N_iso_ distance (Å)	2.408	2.418
*ϕ*, (°)	41.37	44.93
*α*, (°)	54.56	54.60
*r*_ij_, (Å)	8.838	7.892
*θ*, (°)	43	35

## Supplementary Materials

Supplementary materials are available online.
