# Supplementary material for: Molecular Orientation of a Terbium(III)-Phthalocyaninato Double-Decker Complex for Effective Suppression of Quantum Tunneling of the Magnetization
Source: Molecules. 2017 Jun 15;22(6):999. doi: 10.3390/molecules22060999 (PMC6152734; doi:10.3390/molecules22060999)
Supplement: Supplementary file 1 [file molecules-22-00999-s001.pdf]

Molecular Orientation of Terbium(III)-Phthalocyaninato Double-Decker Complex for Effective Suppression of Quantum Tunneling of the Magnetization

Tsutomu Yamabayashi, Keiichi Katoh, Brian K. Breedlove and Masahiro Yamashita

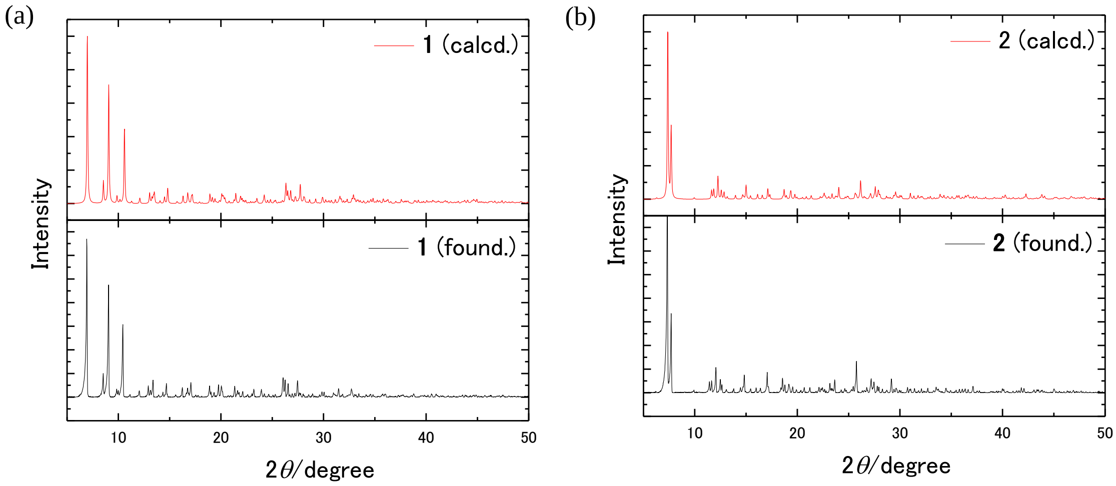

Figure S1. Comparison between experimental and simulated PXRD patterns (5–50°) for **1** and **2**.

The Generalized Debye Model (Equation (S1) and (S2))

$$\chi'(\omega) = \chi_S + (\chi_T - \chi_S)\frac{1+(\omega\tau)^{1-\alpha}\sin(\pi\alpha/2)}{1+2(\omega\tau)^{1-\alpha}\sin(\pi\alpha/2)+(\omega\tau)^{2-2\alpha}} \quad \text{(S1)}$$

$$\chi''(\omega) = (\chi_T - \chi_S)\frac{(\omega\tau)^{1-\alpha}\cos(\pi\alpha/2)}{1+2(\omega\tau)^{1-\alpha}\sin(\pi\alpha/2)+(\omega\tau)^{2-2\alpha}} \quad \text{(S2)}$$

where χ_S is the adiabatic susceptibility, χ_T is the isothermal susceptibility, $\omega = 2\pi\nu$, (ν is the frequency) is the angular frequency, τ is the magnetization relaxation time, and α is the quantitative parameter for the width of the τ distribution.

Arrhenius equation (Equation (S3))

$$\tau = \tau_0 \exp(U_{eff}/k_B T) \quad \text{(S3)}$$

where τ is the magnetization relaxation time, τ_0 is the frequency factor, U_{eff} is the energy barrier for the reversal of the magnetization, and k_B is the Boltzmann constant.

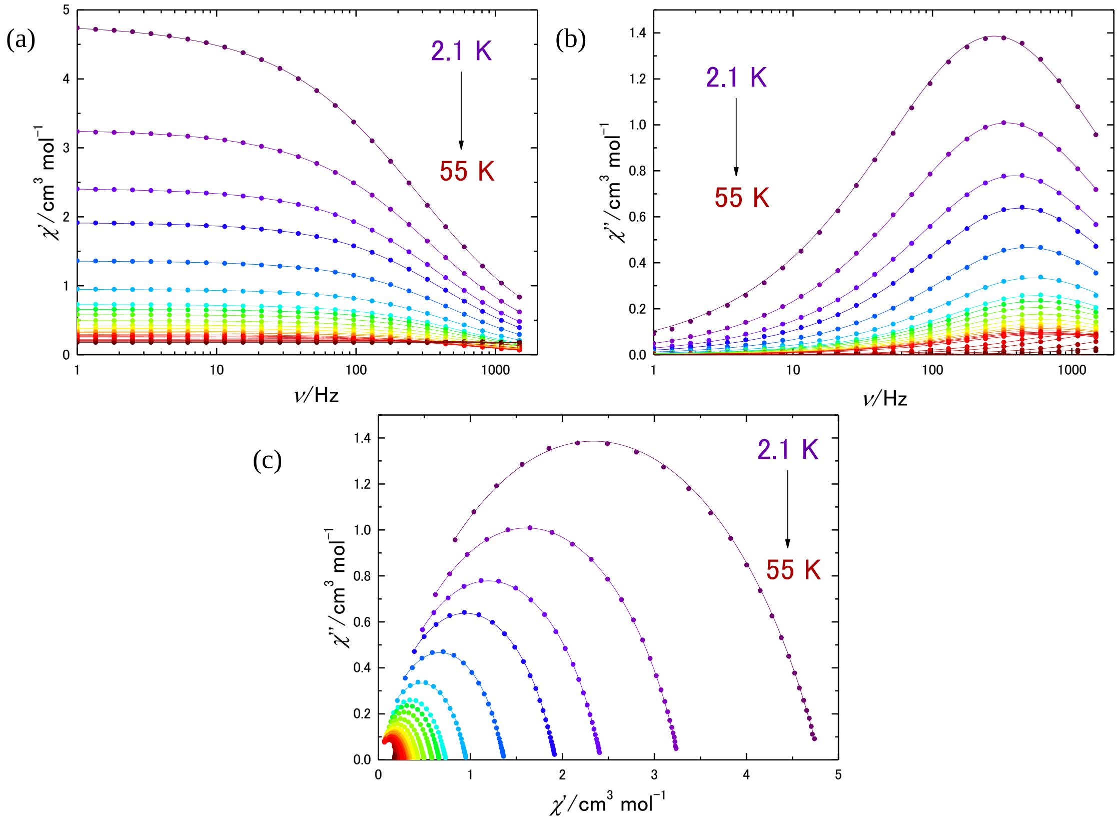

Figure S2. (a) χ_M' and (b) χ_M'' versus ν and (c) Argand plots for **1** in a zero field. The solid lines were fitted by using the generalized Debye model.

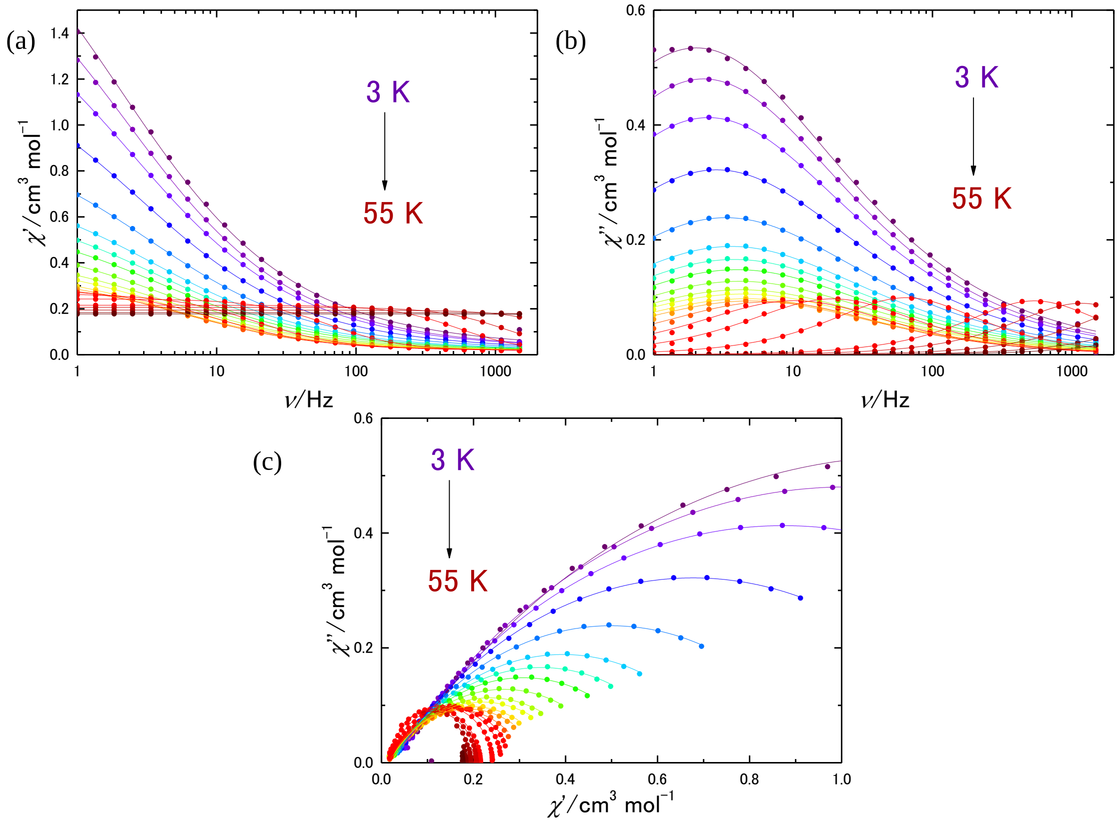

Figure S3. (a) χ_M' and (b) χ_M'' versus ν and (c) Argand plots for **1** in an H_{dc} of 3000 Oe. The solid lines were fitted by using the generalized Debye model.

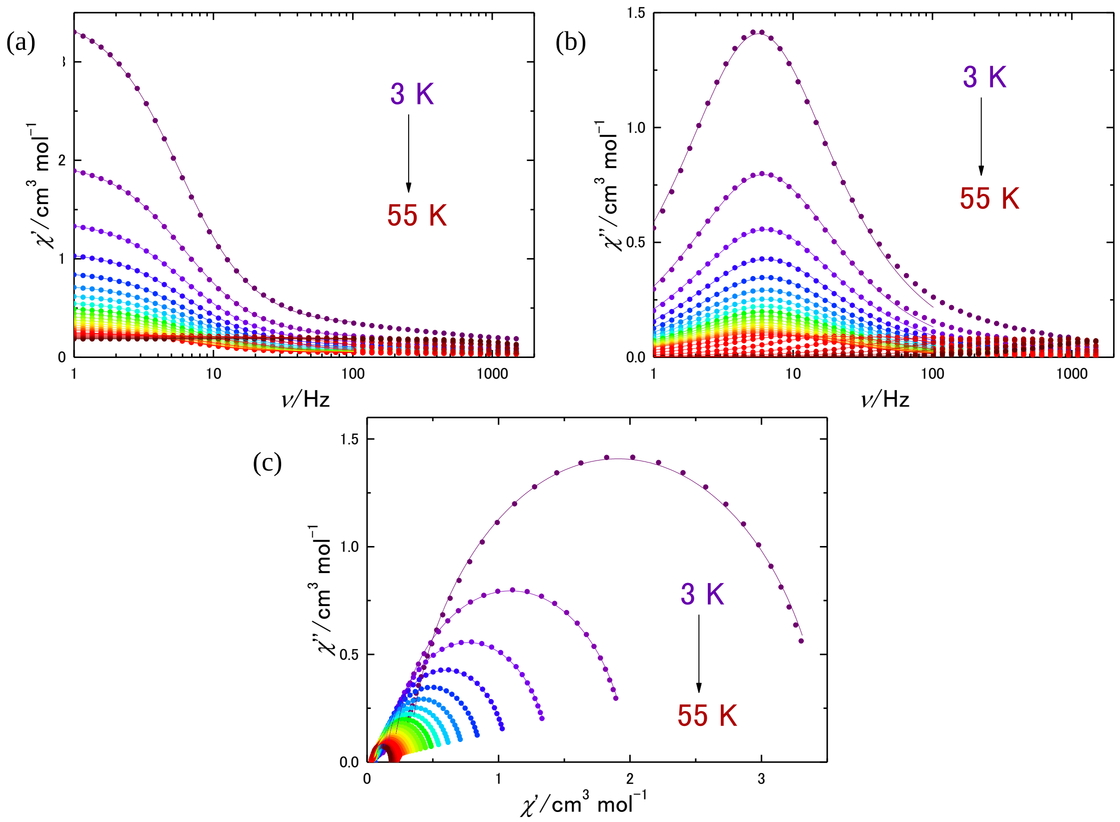

Figure S4. (a) χ_M' and (b) χ_M'' versus ν and (c) Argand plots for **2** in a zero field. The solid lines were fitted by using the generalized Debye model.

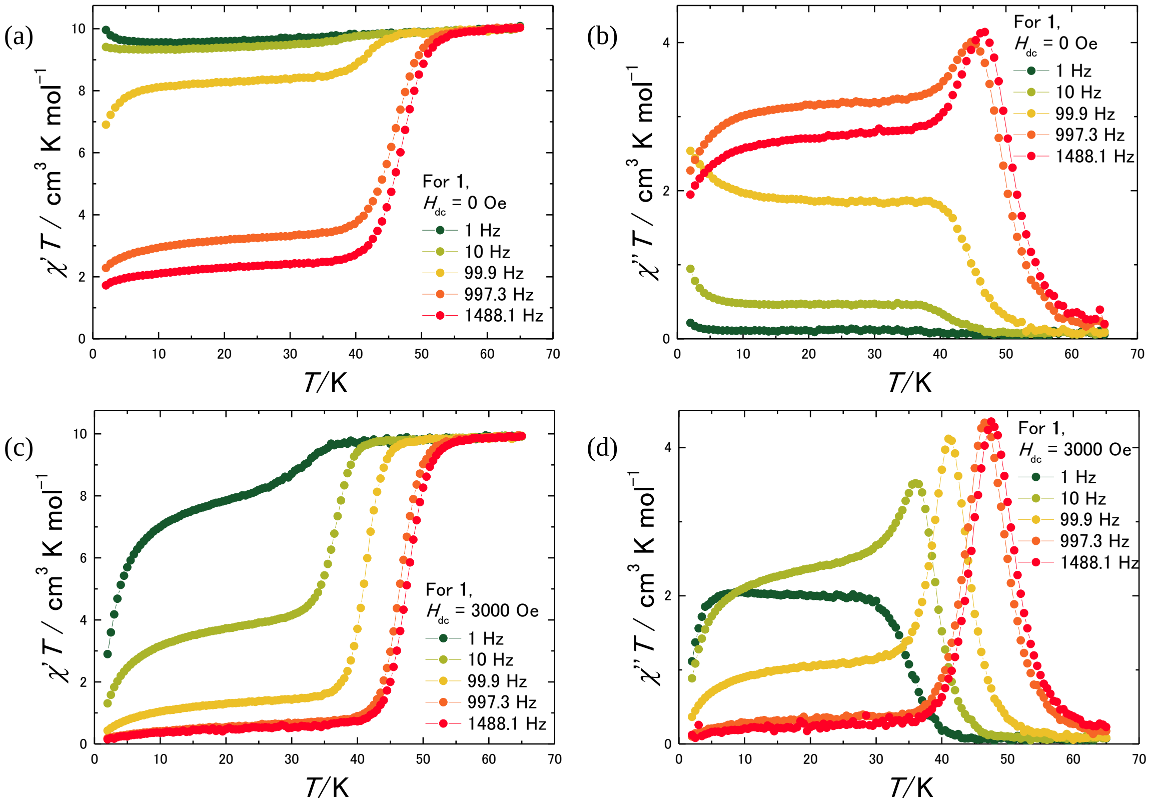

Figure S5. Frequency and temperature dependencies of (a and c) the real and (b and d) imaginary parts of the ac magnetic susceptibilities for **1.** Parts a and b were measured in the absence of a magnetic field, and parts c and d were done in an H_{dc} of 3000 Oe. In all graphs, the solid lines are guides for the eyes.

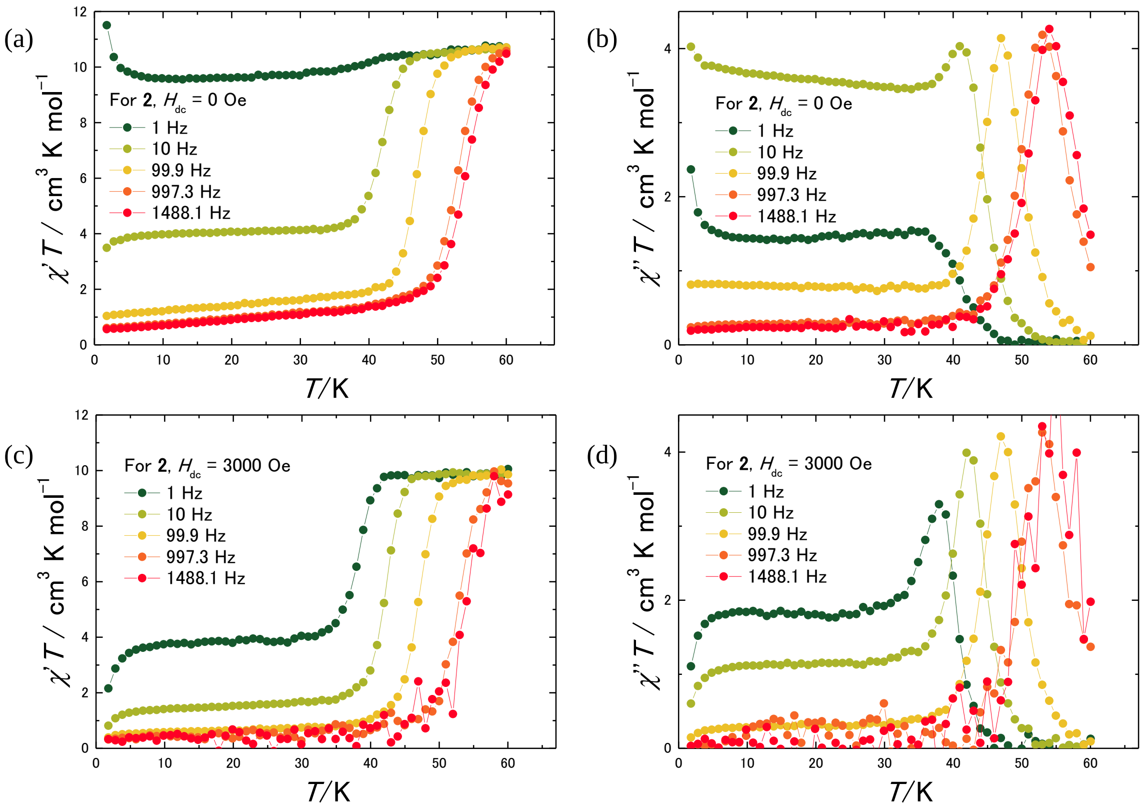

Figure S6. Frequency and temperature dependencies of (a and c) the real and (b and d) imaginary parts of the ac magnetic susceptibilities for **2.** Parts a and b were measured in the absence of a magnetic field, and parts c and d were done in an H_{dc} of 3000 Oe. In all graphs, the solid lines are guides for eyes.

Table S1. Results of fitting for **1**.

T/K	τ @0 Oe/s	τ @3000 Oe/s
3	4.70×10^{-4}	7.88×10^{-2}
4	4.11×10^{-4}	6.98×10^{-2}
5	3.73×10^{-4}	6.45×10^{-2}
7	3.31×10^{-4}	5.63×10^{-2}
10	3.01×10^{-4}	4.84×10^{-2}
13	2.85×10^{-4}	4.40×10^{-2}
14.4	2.80×10^{-4}	4.19×10^{-2}
16.3	2.73×10^{-4}	4.02×10^{-2}
19.2	2.65×10^{-4}	3.85×10^{-2}
22.1	2.64×10^{-4}	3.73×10^{-2}
25	2.58×10^{-4}	3.64×10^{-2}
28	2.58×10^{-4}	3.45×10^{-2}
30	2.51×10^{-4}	3.17×10^{-2}
33	2.51×10^{-4}	2.36×10^{-2}
35	2.49×10^{-4}	1.55×10^{-2}
37	2.38×10^{-4}	8.27×10^{-3}
40	2.34×10^{-4}	2.47×10^{-3}
45	1.36×10^{-4}	2.95×10^{-4}
47	7.42×10^{-5}	1.37×10^{-4}
50	2.97×10^{-5}	4.36×10^{-5}
53	1.59×10^{-5}	7.47×10^{-6}
55	1.06×10^{-5}	3.12×10^{-6}

Table S2. Results of fitting for **2**.

T/K	τ @0 Oe/s
3	2.81×10^{-2}
5	2.58×10^{-2}
7	2.51×10^{-2}
9	2.48×10^{-2}
11	2.48×10^{-2}
13	2.48×10^{-2}
15	2.49×10^{-2}
17	2.51×10^{-2}
19	2.53×10^{-2}
21	2.56×10^{-2}
23	2.58×10^{-2}
25	2.60×10^{-2}
27	2.63×10^{-2}
29	2.65×10^{-2}
31	2.68×10^{-2}
33	2.70×10^{-2}
35	2.69×10^{-2}
39	2.23×10^{-2}
41	1.53×10^{-2}
43	8.19×10^{-3}
45	3.74×10^{-3}
47	1.63×10^{-3}
49	7.22×10^{-4}
51	3.53×10^{-4}
53	1.34×10^{-4}
55	5.85×10^{-5}

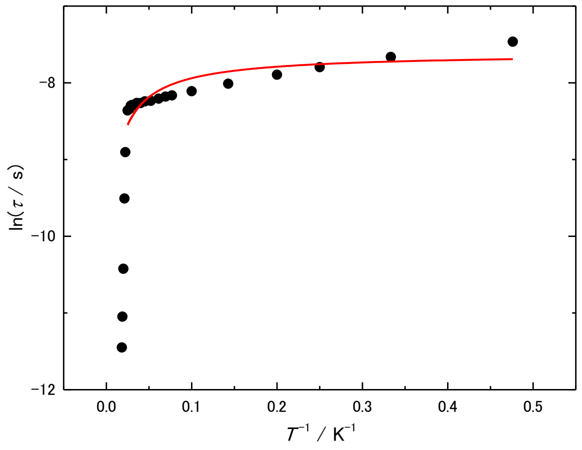

Figure S7. Fitting of the Arrhenius plots for **1** in a zero field considering direct process and QTM using the following equation:

$$\tau = \left\{ AT + \frac{1}{\tau_{QTM}} \right\}^{-1}$$

where the first and second terms represent a direct process and QTM,
A is the coefficients of direct process, and τ_{QTM} is the QTM time
$A = 78.2\ \mathrm{s}^{-1}\mathrm{K}^{-1}$, $\tau_{\mathrm{QTM}} = 4.94 \times 10^{-4}$ s.

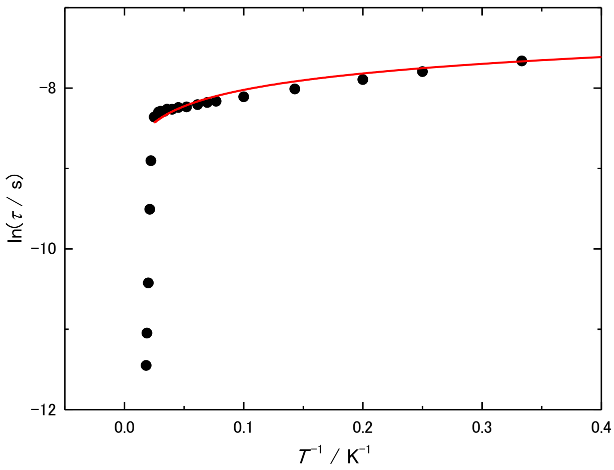

Figure S8. Fitting of the Arrhenius plots for **1** in a zero field considering the Raman process and QTM using the following equation:

$$\tau = \left\{ CT^{n} + \frac{1}{\tau_{QTM}} \right\}^{-1}$$

where the first and second terms represent a Raman process and QTM,
C is the coefficients of Raman process, and *n* is the exponent of the Raman process.
$C = 1548\ \mathrm{s}^{-1}\mathrm{K}^{-n}$, $n = 0.29$, $\tau_{\mathrm{QTM}} = 4.41 \times 10^{22}$ s. As shown in above, the fitting gave meaningless parameters.

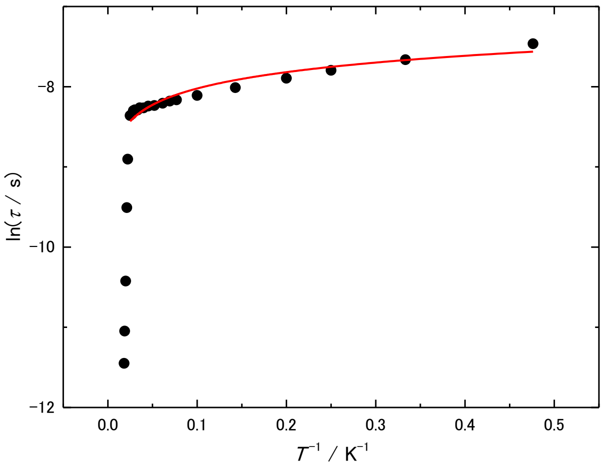

Figure S9. Fitting of the Arrhenius plots for **1** in a zero field considering direct process, Raman process and QTM using the following equation:

$$\tau = \left\{ AT + CT^n + \frac{1}{\tau_{QTM}} \right\}^{-1}$$

A = 5.27 × 10^{-17} $s^{-1}K^{-1}$, C = 1548 $s^{-1}K^{-n}$, n = 0.29, τ_{QTM} = 4.41 × 10^{22} s. As shown in above, the fitting gave meaningless parameters.

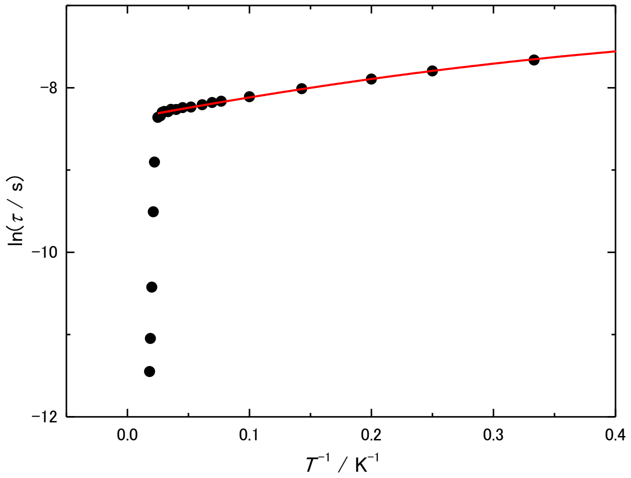

Figure S10. Fitting of the Arrhenius plots for **1** in a zero field considering an Orbach process and QTM using the following equation:

$$\tau = \left\{ \frac{1}{\tau_0} \exp\left(-\frac{U_{eff}}{k_B T} \right) + \frac{1}{\tau_{QTM}} \right\}^{-1}$$

where the first and second terms represent an Orbach process and QTM. U_{eff} = 3.92 cm^{-1}, τ_0 = 3.3 × 10^{-4} s, τ_{QTM} = 7.84 × 10^{-4} s

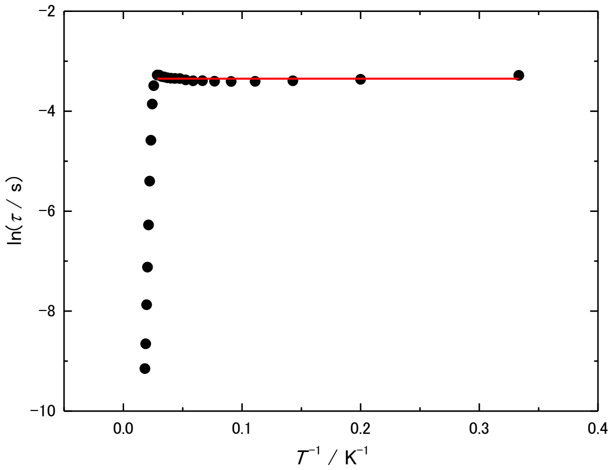

Figure S11. Fitting of the Arrhenius plots for **2** in a zero field considering QTM according to the following equation.

$$\tau = \left\{ \frac{1}{\tau_{QTM}} \right\}^{-1}$$

$\tau_{QTM} = 3.51 \times 10^{-2}$ s.

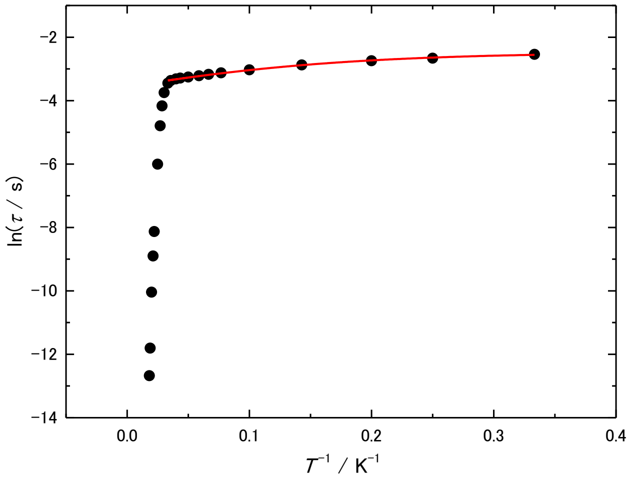

Figure S12. Fitting of the Arrhenius plots for **1** in an H_{dc} of 3000 Oe considering an Orbach process and QTM using the following equation.

$$\tau = \left\{ \frac{1}{\tau_0} \exp\left(-\frac{U_{eff}}{k_B T} \right) + \frac{1}{\tau_{QTM}} \right\}^{-1}$$

$U_{eff} = 9.61$ cm^{-1}, $\tau_0 = 4.2 \times 10^{-2}$ s, $\tau_{QTM} = 8.83 \times 10^{-2}$ s.
